# Matured Tolerogenic Dendritic Cells Effectively Inhibit Autoantigen Specific CD4^+^ T Cells in a Murine Arthritis Model

**DOI:** 10.3389/fimmu.2019.02068

**Published:** 2019-08-28

**Authors:** Manon A. A. Jansen, Rachel Spiering, Irene S. Ludwig, Willem van Eden, Catharien M. U. Hilkens, Femke Broere

**Affiliations:** ^1^Division of Immunology, Department of Infectious Diseases and Immunology, Faculty Veterinary Medicine, Utrecht University, Utrecht, Netherlands; ^2^Musculoskeletal Research Group, Institute of Cellular Medicine, Newcastle University, Newcastle upon Tyne, United Kingdom; ^3^Arthritis Research UK Rheumatoid Arthritis Pathogenesis Centre of Excellence (RACE), Newcastle upon Tyne, United Kingdom; ^4^NIHR-Newcastle Biomedical Research Centre in Ageing and Long-Term Conditions, Newcastle upon Tyne Hospitals NHS Foundation Trust and Newcastle University, Newcastle upon Tyne, United Kingdom; ^5^Department of Clinical Sciences of Companion Animals, Faculty Veterinary Medicine, Utrecht University, Utrecht, Netherlands

**Keywords:** arthritis, tolDCs, CD4^+^ T cell, immune modulation, immune tolerance

## Abstract

Tolerogenic dendritic cells (tolDCs) are a promising treatment modality for diseases caused by a breach in immune tolerance, such as rheumatoid arthritis. Current medication for these diseases is directed toward symptom suppression but no real cure is available yet. TolDC-based therapy aims to restore immune tolerance in an antigen-specific manner. Here we used a mouse model to address two major questions: (i) is a maturation stimulus needed for tolDC function *in vitro* and *in vivo* and is maturation required for functioning in experimental arthritis and (ii) can tolDCs modulate CD4^+^ T cell responses? To answer these questions, we compared matured and immature dexamethasone/vitamin D3-generated tolDCs *in vitro*. Subsequently, we co-transferred these tolDCs with naïve or effector CD4^+^ T cells to study the characteristics of transferred T cells after 3 days with flow cytometry and Luminex multiplex assays. In addition, we tested the suppressive capabilities of tolDCs in an experimental arthritis model. We found that tolDCs cannot only modulate naïve CD4^+^ T cell responses as shown by fewer proliferated and activated CD4^+^ T cells *in vivo*, but also effector CD4^+^ T cells. In addition, Treg (CD4^+^CD25^+^FoxP3^+^) expansions were seen in the proliferating cell population in the presence of tolDCs. Furthermore, we show that administered tolDCs are capable to inhibit arthritis in the proteoglycan-induced arthritis model. However, a maturation stimulus is needed for tolDCs to manifest this tolerizing function in an inflammatory environment. Our data will be instrumental for optimization of future tolDC therapies for autoimmune diseases.

## Introduction

Rheumatoid arthritis (RA) is an autoimmune disease characterized by chronic inflammation in the joints which causes cartilage and bone destruction (1). To date, there is no cure available and treatment is directed toward mitigating symptoms (non-steroidal anti-inflammatory drugs) or dampening the immune response (disease modifying anti-rheumatic drugs) (2, 3). These therapies suppress the immune system non-specifically and thus are not completely effective and have side effects. RA, as other chronic inflammatory diseases, is caused by a disbalance in the immune system. Immune tolerance for self-antigens is not maintained which means that autoreactive T cells can attack the body's own cells.

Regulatory T cells (Tregs), mainly CD4^+^ T cells, are able to restore immune tolerance by suppressing effector cells in an antigen-specific manner. In patients with autoimmune disease, it is thought that a subtle change in function or presence of Tregs is involved in the pathogenesis of the disease. However, a lot of controversy exists in this area. Several studies show that the suppressive capacities of Tregs in synovial fluid of RA patients are diminished, while in peripheral blood these capacities are maintained (4–6). Other studies indicate a decrease in Treg numbers (7, 8), which could cause excessive inflammation in RA. Antigen specific Tregs are able to suppress this excessive inflammation by suppressing the immune cells that cause the pathological autoimmune response while leaving protective immunity intact.

As a tool to induce antigen specific Tregs, tolerogenic dendritic cells (tolDCs) can be used. TolDCs are dendritic cells (DCs) that are modulated to become immune tolerance-inducing. Whether a DC is immune stimulatory or immune tolerant mainly depends on the environmental cues the DCs receive determines if they become immunogenic or tolerogenic. By modulating DCs *in vitro*, they can be steered toward an immune tolerant status. Multiple approaches for inducing a tolerogenic function in antigen presenting cells *in vitro* have been described (9–12). We focus on dexamethasone and 1α,25-dihydroxyvitamin D3 modulated DCs (13–15) because this type of tolDCs has recently been tested in a phase I clinical trial in inflammatory arthritis patients (16).

The advantage of tolDCs is that they can be loaded with an antigen to specifically target autoreactive T cells without affecting other immune responses. We have shown previously that loading tolDCs with disease specific-antigen enhances their efficiency compared to non-loaded tolDCs (17). Some groups show therapeutic effects with non-loaded tolDCs (18), but generating non-specific Tregs may give rise to general immune suppression thereby increasing the risk of infections. Furthermore, administering non-loaded tolDCs might not actively induce Tregs but generate T cell anergy, which in turn can suppress excessive Th17 and Th1 responses (19). Since the autoantigen that causes disease in RA is currently unknown, non-loaded tolDC treatment could be an option.

In addition to the issue of antigen loading, the stability of a tolDC is an important issue to address. Since DCs are essential for both tolerance and immunity they are the sentinels of the immune system. It is plausible that non-stimulated tolDC change their phenotype when entering an immune stimulatory environment. Therefore, partial maturation with lipopolysaccharide (LPS) (15, 20), monophosphoryl Lipid A (MPLA, lipid A portion of LPS) (21) or a cytokine cocktail (22) would be preferable. This potentially stabilizes the phenotype of the tolDC and improves antigen presentation and migration (9, 15). A more complete understanding of the working mechanism of tolDCs will contribute to the development of tolDC treatment in the future. For that reason, we aimed to (i) determine the role of maturation in murine dexamethasone and 1α,25-dihydroxyvitamin D3 induced tolDCs and function in arthritis, and (ii) study the effects of tolDCs on CD4^+^ T cell responses.

## Materials and Methods

### Mice

Female Balb/cAnNCrl of 18–20 weeks old were purchased from Charles River laboratories for *in vivo* arthritis experiments. Male Balb/cAnNCrl of 10 weeks old were purchased from Charles River laboratories for co-transfer studies. hPG TCR transgenic (23) mice were bred at the central animal laboratory of Utrecht University, the Netherlands. Both sexes were used as donor mice. Animals were kept under standard conditions of the animal facility and all experiments were approved by the Animal Experiment Committee of Utrecht University (project number AVD108002016467). Mice were randomly divided in control- or treatment groups and all animals were monitored and scored three times a week during the arthritis experiments.

### BMDC Culture

Bone marrow was isolated from the femur and tibia from Balb/cAnNCrl (both male and female) 10–20 weeks old mice and seeded 450.000 fresh cells/ml in 6 wells plates (Corning costar). As culture medium IMDM (Gibco) supplemented with 10% FCS (Bodinco), 100 units/ml penicillin, 100 μg/ml streptomycin and 5 × 10^−5^ M β-mercaptoethanol in the presence of 20 ng/ml GM-CSF (in house produced) was used. On day 2 an equal volume of fresh culture medium containing 20 ng/mL GM-CSF was added, and on day 4/5 20 ng/mL fresh GM-CSF was supplemented to the culture. Tolerogenicity was induced by adding 10^−6^M dexamethasone (Invivogen) and 10^−10^M 1α,25-dihydroxyvitamin D3 (Enzo Life sciences) to the BMDC culture on day 7. Simultaneously with the tolerogenic agents, 10 μg/mL peptide (hPG: ATEGRVRVNSAYQDK) and maturation stimuli (lipopolysaccharide (LPS) 10 ng/mL; Sigma Aldrich or Monophosphoryl Lipid A (MPLA, 10 ng/mL) from Salmonella minnesota R595; Invivogen) were added. After 8 days of culture at 37°C, 5% CO_2_, the BMDCs or tolDCs were harvested for further experimentation. Before co-culture, BMDCs or tolDCs were replated into 24 wells plates (Corning costar). Before injection in co-transfer experiments, BMDCs or tolDCs were thoroughly washed with medium (2x) and PBS (1x) and kept on ice.

### Co-cultures

For co-culture experiments, spleens from mB29b TCR transgenic mice were pooled and CD4^+^ cells were isolated using Dynal bead isolation (Invitrogen) by negatively selecting CD4^+^ T cells with a mixture of the following in house produced antibodies: anti-B220 (RA3-6B2), anti-CD11b (M1/70), anti-MHC-II (M5/114) and anti-CD8 (YTS169). To gain a naïve population, CD25^+^ and CD44^+^ cells were depleted by adding an anti-CD25 antibody (PC61, produced in house from hybridoma ATCC PC61 and purified from supernatants) and an anti-CD44 antibody (IM7.8, kindly provided by Tibor Glant) in predetermined optimal concentrations. The purified naïve CD4^+^ T cells were added to BMDCs in a 10:1 ratio.

### Flow Cytometry and Antibodies

Flow cytometry was performed with FACS Canto II (BD) with monoclonal antibodies CD4-V500 (RM4-5, BD Biosciences), CD25-PerCP-Cy5.5 (PC61.5, eBioscience/Thermofisher), Thy1.1-PerCP-Cy5.5 (HIS51, eBioscience/Thermofisher), FoxP3-eFluor450 (FJK-16s, eBioscience/Thermofisher), CD62L-PE (MEL-14, BD biosciences), NKp46-PE-Cy7 (29A1.4, eBioscience/Thermofisher) and CD3-APC (145-2C11, BD biosciences). Red blood cells were lysed with ACK (Ammonium-Chloride-Potassium) buffer. For BMDC phenotyping the following monoclonal antibodies were used: I-A/I-E Horizon450 (M5/114.15.2, eBioscience/Thermofisher), CD11c APC (N418, eBioscience/Thermofisher), CD86 FITC (GL1, BD biosciences), PD-L1 PE (10F.9G2, Biolegend), IL12p40/70 PE (C15.6, BD biosciences). To identify dead cells the Zombie NIR fixable viability kit (Biolegend) was used.

### Induction of PGIA

Human proteoglycan (hPG) was isolated from human articular cartilage as described (24). To induce arthritis, Balb/c mice were injected twice intraperitoneally with a mixture of 2 mg DDA and 250 μg human proteoglycan with a 21 day interval. Subsequently, mice were randomized among experimental groups, and arthritis scores were determined in a blinded fashion using a visual scoring system based on swelling and redness of paws as described (24). tolDCs (1 × 10^6^ cells in 200 μL PBS) were injected intravenously on day 17.

### Co-transfers

#### Naïve CD4^+^ T Cell Co-transfer

**Figure 1:**
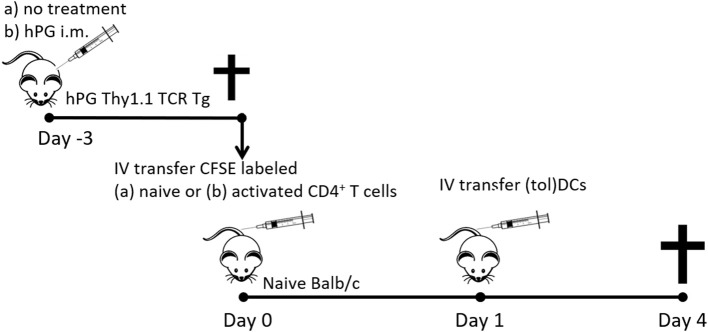
Schematic representation of the co-transfer experiments. Activated hPG Thy1.1^+^ TCR transgenic CD4^+^ T cells where isolated either fromTCR Tg mice that were immunized with hPG i.m. at day −3. Naïve CD4^+^Thy1.1^+^ T cells where isolated from untreated and depleted of CD25^+^ and CD44^+^ CD4^+^ T cells prior to transfer to naïve balb/c mice at day 0. One day after T cell transfer mice received and i.v. injection with (tol)DC as described and after 3 days spleens where isolated for further analysis.

CD4^+^CD25^−^CD44^−^ T cells were purified from spleens of hPG TCR transgenic Thy1.1^+^ mice by Dynal bead isolation (Invitrogen). A representative dot plot of the transferred cells is shown in Supplementary Figure 1A. The purified naïve CD4^+^ T cells (23) were labeled with 0.5 μM 5,6-carboxyfluorescein-succinimidyl-ester (CFSE, Invitrogen) and subsequently intravenously injected (max 10 x 10^6^) in 200 μl PBS in acceptor mice (8–10 weeks old male Balb/cAnNCrl) (day 0). On day one, 1 × 10^6^ freshly cultured hPG pulsed BMDCs or tolDCs were intravenously injected in the same acceptor mice. After 3 days, spleens from acceptor mice were harvested and transferred CD4^+^ T cells were tested for proliferation, activation and phenotype. A schematic prestation of the experiments is depicted in Figure 1.

#### Activated CD4^+^ T Cell Co-transfer

On day −3 the donor hPG TCR transgenic Thy1.1^+^ mice were intra muscularly (left quadriceps) injected with 100 μg hPG peptide in 50 μl PBS (Figure 1). On day 0, CD4^+^ T cells were purified from spleens from hPG TCR transgenic Thy1.1^+^ mice (23) by Dynal bead isolation (Invitrogen). Representative dot plot of the transferred cells and the expression of CD44, CD62L CD25, and FoxP3 are in Supplementary Figure S1B. The purified CD4^+^ T cells were labeled with 0.5 μM 5,6-carboxyfluorescein-succinimidyl-ester (CFSE, Invitrogen) and subsequently intravenously injected in 200 μl PBS in acceptor mice (8–10 weeks old male Balb/cAnNCrl) (day 0). On day one, 2 × 10^5^ or 1 × 10^6^ freshly cultured tolDCs (MPLA stimulated; mtolDC) or BMDCs (LPS matured; mBMDC) were intravenously injected in the same acceptor mice. Both cell types (tolDC or BMDC) were either non-loaded or pulsed with hPG. After 3 days, spleens from acceptor mice were harvested and transferred CD4^+^ T cells were tested on proliferation, activation and phenotype. A schematic prestation of the experiments is depicted in Figure 1.

#### Cytokine Analysis

Supernatants of *ex vivo* stimulations or co-cultures were used for multiplex cytokine analysis of IL-10, IL-2, IL-6, IL-17, and IFN-γ using the Magpix (Luminex XMAP) system according to manufacturer's instructions. Briefly, supernatant together with magnetic beads coated with capture antibodies for the respective cytokines were added to polystyrene, black, 96 wells flat bottom plates (Greiner bio-one, 655096). Subsequently, biotin-conjugated detection antibodies and Streptavidin-PE (BD Bioscience) were added and incubated together. The antibody pairs used:

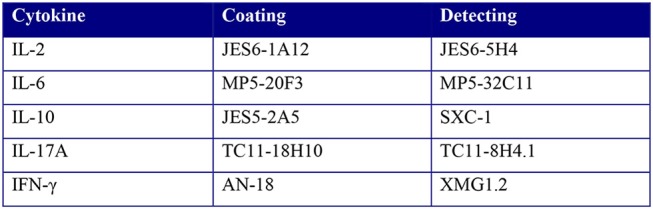



The concentrations of cytokines in the tested samples were calculated using standard curves of recombinant proteins and the MFI data was analyzed using a 5-parameter logistic method (xPONENT software, Luminex, Austin, USA).

### ELISpot

Multiscreen IP filter plate plates (Milipore) were activated with 70% Ethanol and coated with a rat anti-mouse anti-IFN-γ antibody (clone AN-18, in house produced) or anti-IL-10 antibody (JES5-2A5, in house produced) at 2 μg/ml in PBS and then blocked with IMDM medium supplemented with 5% FCS. Subsequently, single cell suspensions of spleen from co-transferred acceptor mice were cultured in 200 μl complete medium for 48 h in 96-wells flat bottom plates for ELISPOT (Millipore) at 5 × 10^6^ cells/ml. Medium or 20 μg/ml hPG peptide were added as restimulation. After culture, the plates were washed and the IFN-γ or IL-10 producing cells were detected using the rat anti-mouse biotin-anti-IFN-γ antibody (clone XMG1.2, BD biosciences) or rat anti-mouse biotin-anti-IL-10 antibody (clone SXC-1, BD biosciences), respectively. Streptavidin-alkaline phosphatase (Sigma, S2890) and BCIP/NPT solution (Roche) were used to visualize the spots according to manufacturer's instructions. Counting of the IFN-γ or IL-10 producing cells was done by the Automated ELISpot Assay Video Analysis System (A.EL.VIS GmbH). To calculate the relative increase (stimulation index: SI), the negative (medium) control was set to 1. The stimulated conditions (hPG or αCD3) were calculated relative to the control: (hPG/αCD3 sample x 1)/ negative control.

### Statistical Analyses

The following statistical analyses were performed using Prism 7.04: repeated measures analysis of variance (ANOVA) with Dunnett or Bonferroni correction for comparisons between multiple groups, paired Student *t*-test for comparisons between two groups.

## Results

### Peptide Pulsed tolDCs Show a Semi-mature Phenotype and Hamper CD4^+^ T Cell Activation *in vitro*

**Figure 2:**
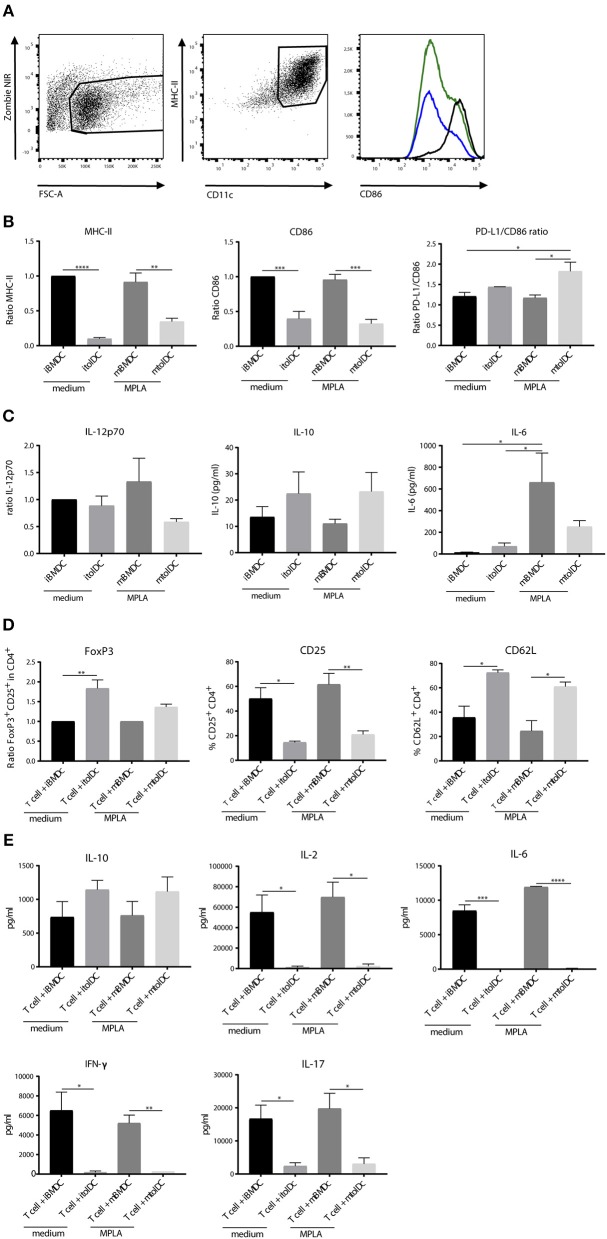
TolDCs exhibit a semi-mature phenotype and remain stable after challenge *in vitro*. TolDCs were generated by adding dexamethasone and 1,25-dihydroxyvitamin D3 and stimulated with MPLA or medium as control. Phenotype was measured by flow cytometry. In the histogram, mBMDCs (black line), itolDCs (blue) and mtolDCs (green) representatives are shown **(A)**. The ratio to immature BMDC was used to determine the difference in expression of MHC-II, CD86 and PD-L1 in the CD11c^+^ population. The ratio was used for MHC-II and CD86 because of the inter-experimental variance. iBMDCs were set to 1 and mBMDC and tolDCs were compared to these cells **(B)**. Cytokine production **(C)** was measured in the supernatant by Magpix (IL-10, IL-6) or intracellular by flow cytometry (IL-12p70). iBMDCs, mBMDCs, itolDCs or mtolDCs were pulsed with peptide and co-cultured for three days with naïve (CD25^+^ and CD44^+^ depleted) CD4^+^ T cells. On day 3, phenotype of the CD4^+^ T cells was determined by flow cytometry **(D)**. For CD25+FoxP3, the ratio (in CD4^+^ population) to CD4^+^ T cells that were in co-culture with BMDCs was used to compare the difference when co-culturing with tolDCs. The ratio was used because of the inter-experimental variance. The respective control BMDC-T cell co-cultures were set to 1 and tolDC co-cultured T cells were compared to BMDC co-cultured T cells. As markers for activation status of the CD4^+^ T cell, CD25 and CD62L were measured. Cytokine production was measured after co-culture in the supernatant by Magpix **(E)**. Data shown are mean ± SEM. Two-tailed paired student *T*-test was used. *p ≤ 0.05, **p ≤ 0.01, ***p ≤ 0.001, ****p ≤ 0.0001. *N* = 4.

First, we investigated the change in phenotype of tolDCs after stimulation with a TLR4 agonist *in vitro*. DCs were generated from bone marrow and treated with dexamethasone and the active form of vitamin D3 to develop tolDCs. Untreated bone marrow derived dendritic cells (BMDCs) were used as controls. To compare the phenotype of BMDCs and tolDCs, both cell types were measured unstimulated (immature BMDCs; iBMDCs or immature tolDCs; itolDCs) or after stimulation with the lipid A portion of LPS: MPLA (mature BMDCs; mBMDCs or mature tolDCs; mtolDCs). TolDCs were stimulated with MPLA since TLR4 stimulation might be required for antigen-processing and migration. Considering that LPS cannot be used in humans, we used MPLA. Both itolDCs and mtolDCs exhibited a semi-mature phenotype, consisting of a lower expression of MHC-II, CD86 (Figures 2A,B) and a higher PD-L1/CD86 ratio compared to their respective control BMDCs (iBMDCs or mBMDCs). The PD-L1/CD86 ratio is considered indicative of a tolerogenic phenotype as described in multiple studies (20, 25, 26) Furthermore, MPLA stimulation of tolDCs did not induce a proinflammatory cytokine profile as shown by the trend toward a higher IL-10 production and lower IL-12 and IL-6 (Figure 2C) which is important since cytokine signaling is one of the mechanisms of DCs to communicate. To study their function, peptide pulsed itolDCs or mtolDCs were co-cultured with naïve CD4^+^ T cells from a TCR transgenic mouse (27). After 3 days of co-culture with itolDCs or mtolDCs, fewer CD4^+^ T cells expressed the activation marker CD25. Additionally, more CD4^+^ T cells expressed CD62L compared to the controls. Next to this, there was a trend toward more CD4^+^FoxP3^+^ cells after co-culturing with tolDCs (Figure 2D, Supplementary Figure S2). To correct for inter experimental variance in FoxP3% data have been normalized and represented as ratio compared to iBMDC treated mice. CD4^+^ T cells that were in co-culture with non-peptide pulsed itolDCs or mtolDCs did not show any activation or proliferation since these CD4^+^ T cells are antigen-specific. To address CD4^+^ T cell activation and differentiation status their cytokine profile was analyzed (Figure 2E). TolDCs impair pro-inflammatory cytokine production of the CD4^+^ T cells by 2–20 fold compared to CD4^+^ T cells stimulated by control BMDC. In addition the anti-inflammatory cytokine IL-10 production was not diminished shifting the cytokine balance/profile toward a tolerogenic profile in co-culture with tolDCs, indicating that these T cells contain a more regulatory character. Thus, tolDCs modulate CD4^+^ T cell activation and drives them toward an immune tolerant state. In these experiments, maturation of the tolDCs did not influence their effect on CD4^+^ T cells.

### TolDCs Restrict the Activation of Naïve Antigen Specific CD4^+^ T Cells *in vivo*

**Figure 3:**
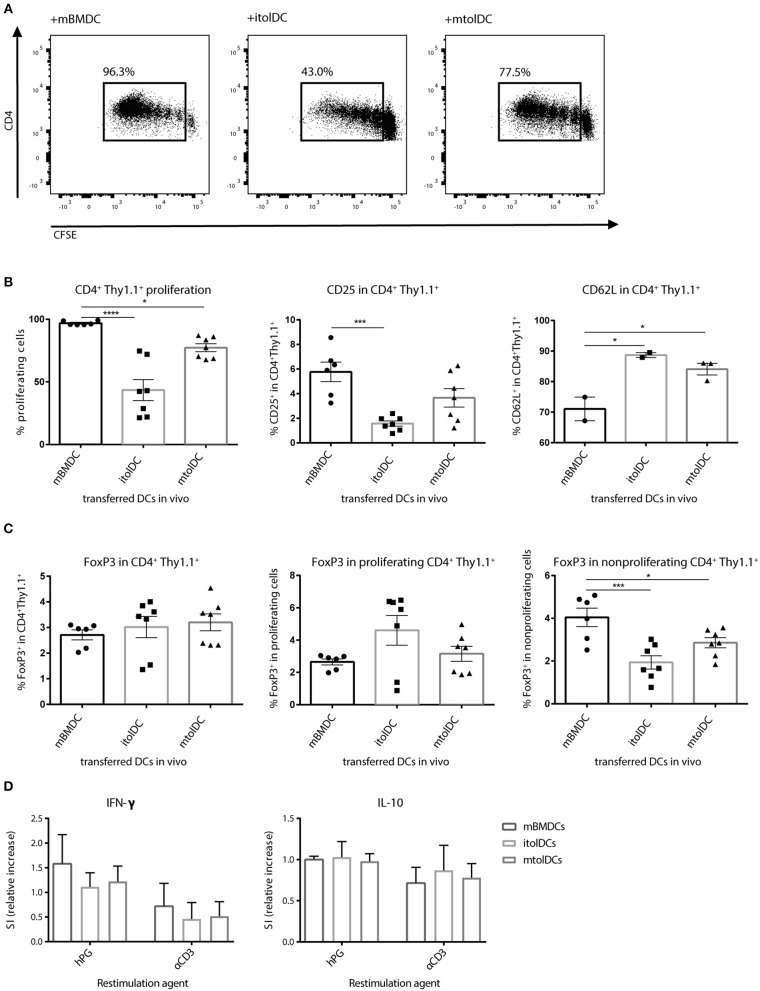
tolDCs restrict the activation of naïve antigen specific CD4^+^ T cell *in vivo*. (Im)mature tolDCs or mature BMDCs were pulsed with hPG peptide and transferred (1 × 10^6^ cells/ injection) 1 day after the naïve CFSE labeled hPG TCR transgenic CD4^+^ T cells. Proliferation **(A,B)** and phenotype **(B,C)** from the transferred CD4^+^Thy1.1^+^ T cells was measured by flow cytometry. To further examine the activation status of the transferred CD4^+^ Thy1.1^+^ T cells, splenocytes were stimulated with hPG (antigen specific) or soluble αCD3 (general). After 3 days, IFNγ and IL-10 producing cells were measured by ELISpot **(D)**. Data shown are mean ± SEM. Each dot represents an individual mouse. Data are from three independent experiments. One way ANOVA (Dunnett) was used. *p ≤ 0.05, ***p ≤ 0.001, ****p ≤ 0.0001.

To determine if tolDCs can affect naïve CD4^+^ T cells *in vivo* we performed co-transfer experiments. First, we transferred naïve hPG-TCR Thy1.1^+^ transgenic CD4^+^ T cells and subsequently (1 day later) itolDCs or mtolDCs pulsed with hPG peptide into a naïve acceptor mouse. By transferring CD4^+^ T cells that contain a congenic marker (Thy1.1), we were able to select only the transferred CD4^+^ T cells for analysis *ex vivo*. As a control, LPS matured BMDCs (mBMDCs) instead of tolDCs loaded with hPG peptide were transferred with naïve CD4^+^ T cells. Nearly 100% of the CD4^+^ Thy1.1^+^ T cells that were co-transferred with mBMDCs proliferated, while the CD4^+^Thy1.1^+^ T cells that were co-transferred with itolDCs or mtolDCs proliferated, respectively 45 and 75% (Figures 3A,B). At the same time, the CD25 expression was lower on the hPG TCR transgenic CD4^+^Thy1.1^+^ T cells that were co-transferred with itolDCs. Furthermore, the antigen specific CD4^+^ Thy1.1^+^ T cells that were in the presence of itolDCs or mtolDCs showed a lower CD62L expression compared to the control (Figure 3B). This indicates that next to *in vitro* modulation, both immature and mature tolDC can also modulate the CD4^+^ T cell response *in vivo*. The percentage CD4^+^FoxP3^+^ in the total CD4^+^Thy1.1^+^ transferred cells was not significantly increased when tolDCs were co-transferred. However, in the presence of itolDCs, we observed a trend toward a higher percentage CD4^+^FoxP3^+^ cells in the proliferating population (Figure 3C and Supplementary Figure S3).

To further define the phenotype of the CD4^+^ T cells *ex vivo*, IL-10 and IFN-γ secreting cells were measured by ELISpot. The amount of IL-10 producing antigen specific CD4^+^ T cells was similar when itolDCs, mtolDCs, or mBMDCs were present *in vivo*, and although not significantly visually the amount of IFN-γ producing cells tended to be lower when mice were co-transferred with itolDCs or mtolDCs (as shown by the decreasing trend in Figure 3D). These results confirm that CD4^+^ T cell activation is constrained after encountering tolDCs *in vivo*.

### TolDCs Are Capable of Modifying Effector CD4^+^ T Cells

**Figure 4:**
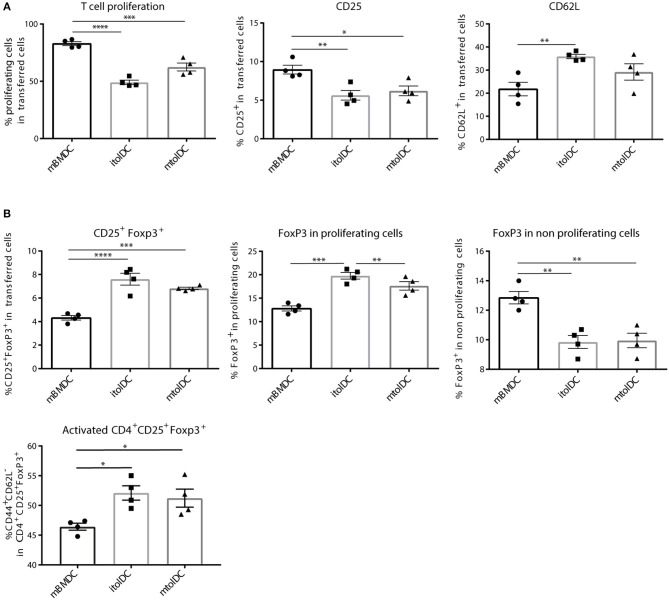
tolDC pulsed with peptide inhibit further activation of CD4^+^ T cells *in vivo*. First, hPG TCR transgenic CD4^+^Thy1.1^+^ T cells were activated *in vivo* by injecting hPG peptide i.m. into the transgenic mice. After 3 days, CD4^+^ Thy1.1^+^ TCR transgenic T cells were isolated and transferred into a naïve Balb/c acceptor mouse. One day later, hPG pulsed itolDC, mtolDC or mBMDCs were injected into the acceptor mice. After 3 days, phenotype and proliferation of the transferred CD4^+^ Thy1.1^+^ T cells was measured by flow cytometry in spleen **(A,B)**. Data shown are mean ± SEM. Each dot represents an individual mouse. One way ANOVA (Dunnett) was used. *p ≤ 0.05, **p ≤ 0.01, ***p ≤ 0.001, ****p ≤ 0.0001.

Since tolDCs are intended to be used as therapeutic agents under inflammatory conditions, restraining the activation of naïve CD4^+^ T cells alone may not be sufficient. In addition, tolDCs should also be able to diminish responses of activated T cells. Therefore, we performed co-transfer studies with a mixture of (both memory and effector) activated CD4^+^ T cells to investigate if tolDCs are capable to modify such proinflammatory T cells. We co-transferred itolDC, mtolDC or mBMDC pulsed with peptide and activated peptide-specific CD4^+^Thy1.1^+^ T cells into a naïve acceptor Balb/c mouse. Both itolDCs and mtolDCs inhibited further proliferation and activation of the peptide-specific CD4^+^Thy1.1^+^ T cells *in vivo* (Figure 4A), similar as was seen when naïve CD4^+^Thy1.1^+^ T cells were injected. Next to this, in mice that were injected with tolDCs, not only more Tregs (CD4^+^CD25^+^FoxP3^+^) were measured (Figure 4B, Supplementary Figure S4), but these CD4^+^CD25^+^FoxP3^+^ cells were also more activated as shown by an increase in CD44 expression when compared to mice that received mBMDCs (Figure 4B). In addition, the spleens of mice which received tolDCs contained more naïve CD4^+^Thy1.1^+^ T cells 3 days after co-transfer as shown by an increase in CD62L positive cells and a decrease in CD25 (Figure 4A) and CD44 (data not shown). These results indicate that tolDCs are not only able to modulate naïve CD4^+^ T cells but also previously activated CD4^+^ T cells *in vivo*. Mice in which activated CD4^+^ T cells were co-transferred with itolDC, mtolDC or mBMDC pulsed with an irrelevant peptide did not show any proliferation or activation of the peptide-specific CD4^+^ T cells, showing that these are antigen dependent (data not shown).

### TolDCs Ameliorate Proteoglycan Induced Arthritis

**Figure 5:**
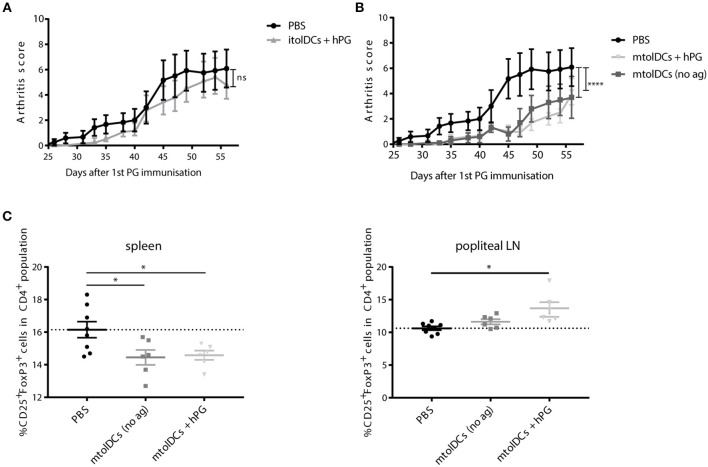
hPG loaded or non-loaded tolDCs matured with MPLA ameliorate PGIA. Arthritis was induced by injecting the mice two times (day 0 and day 21) with hPG protein and DDA. TolDCs (1 × 10^6^ cells in 200 μL PBS) were administered intravenously on day 17, before the second hPG/DDA injection. Mice were scored three times a week for arthritis. The arthritis scores are based on a visual scoring system. Mice received either PBS, immature tolDCs (itolDC) **(A)** or mtolDCs **(B)** non-loaded or pulsed with disease specific antigen (hPG). Data shown in A and B are from one *in vivo* experiment. PBS: *n* = 6, mtolDCs no ag: *n* = 5, mtolDCs + hPG: *n* = 7 **(C)** The percentage CD4^+^CD25^+^FoxP3^+^ (Treg) was analyzed *ex vivo* in the spleen and popliteal lymph nodes by flow cytometry. Data shown are mean ± SEM. Each dot represents an individual mouse. Two way ANOVA (Dunnett) was used to compare all three groups in an unbiased fashion. *p ≤ 0.05, ****p ≤ 0.0001.

To study if tolDCs are able to affect arthritic symptoms, we performed *in vivo* arthritis studies in the proteoglycan induced arthritis (PGIA) model. Female Balb/c mice were injected with hPG protein and dimethyl-dioctadecylammonium (DDA) two times intraperitoneal with a 3-week interval to induce arthritis. Non-loaded tolDCs or tolDCs loaded with hPG peptide were injected intravenously on day 17 in the pre-clinical phase of disease. As pre-clinical phase is considered the stage in which the mice received the first immunization with hPG protein and DDA. In these 2 weeks before the second hPG/DDA injection, the mice develop antibodies against hPG but do not experience symptoms yet (28). This is comparable to humans in whom autoantibodies can be detected years before symptoms occur. Mice treated with itolDCs showed equal arthritis scores compared to the PBS mice (Figure 5A). However, treatment with non-loaded mtolDCs as well as mtolDCs loaded with hPG resulted in a reduced and delayed development of arthritis (Figure 5B). No significant difference was observed between mice that were injected with non-loaded mtolDCs or mtolDCs loaded with hPG peptide. These results indicate that a maturation stimulus is needed for tolDCs to be effective *in vivo*. Taking the day of onset and maximum arthritis score into account (Table 1, data combined from two independent *in vivo* studies), the mice that were treated with mtolDCs not only show less arthritis but develop symptoms at a later time point than PBS mice.

**Table 1:** MtolDCs ameliorate PGIA and delay the onset of disease.

**Group**	**Day of onset**	**Maximum arthritis score**	**Total number of mice (*n*)**
**PBS**	35.36 ± 8.0	5.82 ± 3.52	14
**mtolDCs (no ag)**	41.27 ± 7.45	3.36 ± 3.44	11
**mtolDCs** **+** **hPG**	41.85 ± 7.49	3.269 ± 2.48	13

*MtolDCs reduce arthritis scores (maximum arthritis scores) and delay the onset of PGIA (day of onset) when compared to the PBS mice. As day of onset is considered the first day that a mouse has a score of 1. Two-tailed paired student T-test was used, no significant differences*.

*Ex vivo* analyses of spleen and draining lymph nodes (popliteal lymph nodes) show that Tregs (defined as CD4^+^CD25^+^FoxP3^+^ cells) are present in lower quantities in the spleen (Figure 5C) in mtolDC treated mice as compared to PBS mice (controls). However, in the popliteal lymph node there are more CD4^+^CD25^+^FoxP3^+^ cells present only in mice that received antigen-pulsed mtolDCs compared to PBS mice (Figure 5C, Supplementary Figure S5). The arthritis experiments described show that tolDC treatment can be effective if tolDCs are stimulated with MPLA prior to infusion.

### Matured tolDCs Are Able to Take Up Antigen *in vivo*

We observed in the arthritis experiments (Figure 5) that non-loaded mtolDCs are also able to lower arthritic symptoms. To investigate if this was due to general immunosuppressive factors (e.g. cytokines) or antigen dependent, we performed a co-transfer experiment in which we tested if mtolDCs are able to take up hPG *in vivo*. Therefore, hPG TCR transgenic CD4^+^Thy.1.1^+^ T cells were injected into an acceptor mice followed 1 day later by an intramuscular injection with hPG peptide and an intravenous injection with mtolDCs or mBMDCs. To study if non-pulsed mtolDCs could internalize hPG *in vivo*, we included a condition in which the acceptor mice received no DC injection but only the hPG TCR transgenic CD4^+^Thy1.1^+^ T cells.

**Figure 6:**
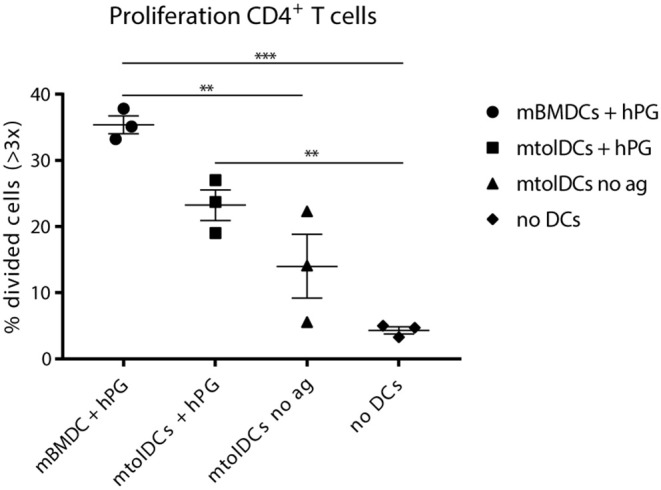
Non-loaded mtolDCs take up hPG *in vivo*. First, CD4^+^ TCR transgenic T cells were isolated and transferred into a naïve Balb/c acceptor mouse 1 day later, hPG peptide was injected i.m into the acceptor mouse followed by i.v. injection of non-loaded mtolDCs or hPG pulsed mtolDCs or mBMDC. After 3 days, proliferation of the transferred CD4^+^ T cells was measured by flow cytometry). To minimize background of spontaneous proliferation, cells that have divided more than 3 times (>3x) were analyzed. Data shown are mean ± SEM. Each dot represents an individual mouse. *N* = 3 per group. One way ANOVA (Dunnett) was used. **p ≤ 0.01, ***p ≤ 0.001.

The mice that received no intravenous (tol)DC injection show 3–5% CD4^+^ T cells that proliferated adequately (Figure 6). This is most likely proliferation induced by APCs from the recipient mouse presenting hPG. The mice that were injected with non-loaded mtolDCs (“mtolDCs no ag” in Figure 6) show 5–22% proliferating CD4^+^ T cells. This indicates that the non-pulsed mtolDCs indeed are able to take up hPG *in vivo* and actively presented it to CD4^+^ T cells. The mice treated with hPG loaded mBMDCs and mtolDCs show similar responses as in Figures 2, 3.

## Discussion

TolDCs are potentially useful for inducing immune tolerance in RA. In this study we addressed two questions: (i) is tolDC maturation required for their function *in vitro* and *in vivo*, and (ii) can tolDCs modulate CD4^+^ T cell responses. TolDCs are able to hamper activation and proliferation of naïve and effector CD4^+^ T cells *in vitro* or under immune homeostatic circumstances. However, tolDC maturation is required under inflammatory conditions to inhibit arthritis.

Since maturation of tolDCs induces metabolic changes in the DC which might be necessary for stabilization, cell survival and function of tolDCs (9, 15, 29), we tested both non-stimulated and MPLA stimulated tolDCs. Furthermore, we also tested LPS stimulated tolDCs on phenotype and functionality which gave similar results to MPLA stimulated tolDC (Supplementary Figure S6). In addition, mtolDC phenotype remained stable even in the presence of a pro-inflammatory cytokine mix consisting of IL-1β, GM-CSF, IL-6, TNF, and IFNγ *in vitro* (data not shown).

As shown in Figure 5, tolDCs need stimulation with MPLA to exert their function in the arthritis model. However, itolDCs were also efficient in modulating the CD4^+^ T cell response in the co-transfer studies. We hypothesize that this difference is caused by the proinflammatory milieu in the arthritis experiments, which is not present in the *in vivo* co-transfer experiments. itolDCs are able to modulate CD4^+^ T cells in these “neutral” environments, but when a proinflammatory milieu is present they are not potent enough to induce tolerance and/or disease suppressing responses or cannot reach the inflammatory sites due to a lack of migration.

To answer the second question, if tolDCs can modulate CD4^+^ T cells, we investigated how tolDCs influence CD4^+^ T cells to adopt a tolerant phenotype. As shown in the co-transfer studies, tolDCs abrogated not only the activation and proliferation of naïve CD4^+^ T cells (Figure 3) but also inhibited further activation of pre-activated CD4^+^ T cells (Figure 4). Next to effects on CD4^+^ T cell activation, we have shown that tolDCs also affect CD4^+^CD25^+^FoxP3^+^ cells (Tregs). The observation locally enhanced numbers of CD25^+^FoxP3^+^ are present (Figure 5C) can imply that Tregs migrate to the site of inflammation under the influence of mtolDCs, however intrinsic differences in Treg homeostasis in the different lymphoid organs cannot be excluded (30). Furthermore, the co-transfer studies indicate that tolDCs have the potential to activate Tregs (Figure 4).

Currently, there is still a lot of debate about antigen loading of tolDCs. In our experiments antigen loading did not significantly enhance the efficacy of treatment. Both the non-loaded mtolDC as the hPG loaded mtolDC injections caused reduced and delayed arthritis symptoms in the treated mice (31). However, this antigen-independence might be evoked by the fact that the mtolDCs were administered before the second injection with hPG/DDA. A possible explanation that the non-loaded mtolDCs reduced arthritis, is that they took up hPG *in vivo* since by injecting hPG/DDA a depot is formed thereby inhibiting arthritis in an antigen-specific manner. This hypothesis is supported by the data shown in figure 6. In addition, preliminary studies have shown that if we add supernatant from cultured tolDCs to antigen presenting cells in the presence of CD4^+^ T cells *in vitro*, the proliferation of the CD4^+^ T cells is abrogated (unpublished observations), indicating that the tolDCs exert their function, at least partly, via soluble mediators (e.g., cytokines).

In conclusion, tolDCs abrogate activation and proliferation of both naïve as pre-activated CD4^+^ T cells and potentially activate Tregs. Although itolDCs are effective under immune homeostasis, maturation of the tolDCs is needed to inhibit experimental arthritis.

## Ethics Statement

All experiments were approved by the Animal Experiments Committee of Utrecht University (project number AVD108002016467).

## Author Contributions

MJ designed and performed experiments, analyzed data, wrote the paper, and approved the submitted version. RS and CH analyzed data, commented on the manuscript at all stages, and approved the submitted version. IL performed experiments, commented on the manuscript at all stages, and approved the submitted version. WvE commented on the manuscript at all stages and approved the submitted version. FB designed experiments, analyzed data, commented on the manuscript at all stages, and approved the submitted version.

### Conflict of Interest Statement

WvE has shares in Trajectum Pharma, Inc., a SME that develops HSP peptides for immunotherapy. The remaining authors declare that the research was conducted in the absence of any commercial or financial relationships that could be construed as a potential conflict of interest.
