# Supplementary material for: Matured Tolerogenic Dendritic Cells Effectively Inhibit Autoantigen Specific CD4+ T Cells in a Murine Arthritis Model
Source: Front Immunol. 2019 Aug 28;10:2068. doi: 10.3389/fimmu.2019.02068 (PMC6724516; doi:10.3389/fimmu.2019.02068)
Supplement: Supplementary file 1 [file Data_Sheet_1.docx]

**Supplementary information**

**Matured tolerogenic dendritic cells effectively inhibit autoantigen specific CD4+ T cells in a murine arthritis model**

Manon A.A. Jansen, Rachel Spiering, Irene S. Ludwig, Willem van Eden, Catharien M.U. Hilkens, Femke Broere

**
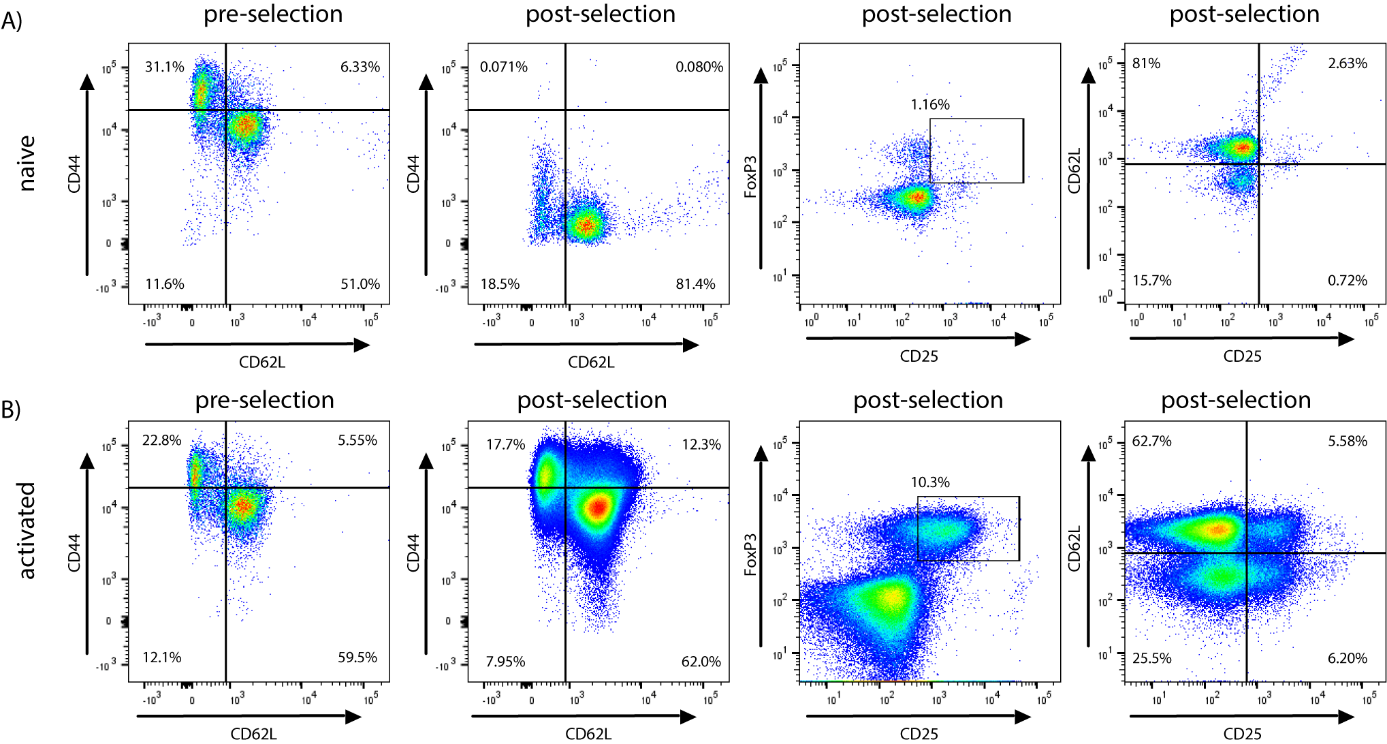
**

**Supplementary Figure S1.**

*Phenotype of transferred T cells.*

After isolation of CD4^+^ T cells from spleen obtained from hPG TCR transgenic mice, they were adoptively transferred to naïve Balb/c acceptor mice. To generate naïve CD4^+^ T cells CD25^+^ and CD44^+^ cells were depleted. The naivety of the cells is especially shown in the CD62L/CD44 plot **(A)**. If these activated cells were not depleted, we considered them activated CD4^+^ T cells **(B)**.

**
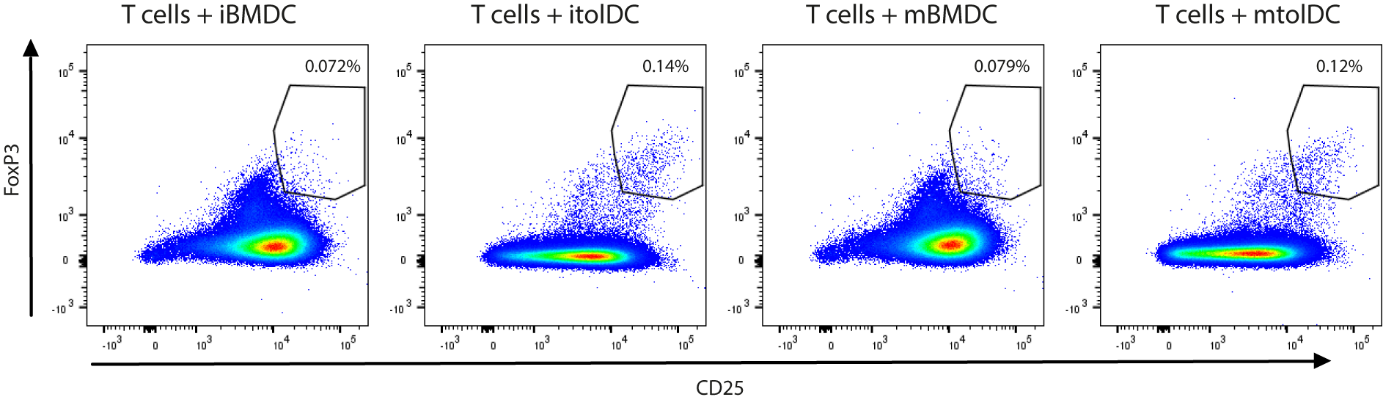
**

**Supplementary Figure S2.**

TolDCs were generated by adding dexamethasone and 1,25-dihydroxyvitamin D3 and stimulated with MPLA or medium as control. iBMDCs, mBMDCs, itolDCs or mtolDCs were pulsed with peptide and co-cultured for three days with naïve (CD25^+^ and CD44^+^ depleted) CD4^+^ T cells. On day 3, phenotype of the CD4^+^ T cells was determined by flow cytometry. Expression of CD25+ and FoxP3+ after coculture in CD4^+^ population was determined. Data are representative dot plots of 4 independent experiments.

**
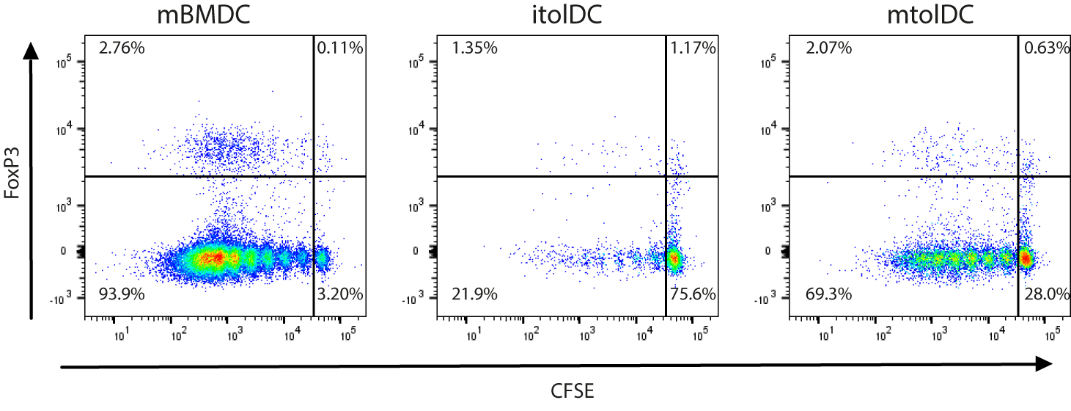
**

**Supplementary Figure S3.**

(Im)mature tolDCs or mature BMDCs were pulsed with hPG peptide and transferred (1x106 cells/ injection) one day after the naïve CFSE labeled hPG TCR transgenic CD4+ T cells. Representative plots of CSFE dilution in and FoxP3 expression by the transferred CD4+Thy1.1+ T cells are shown.

**
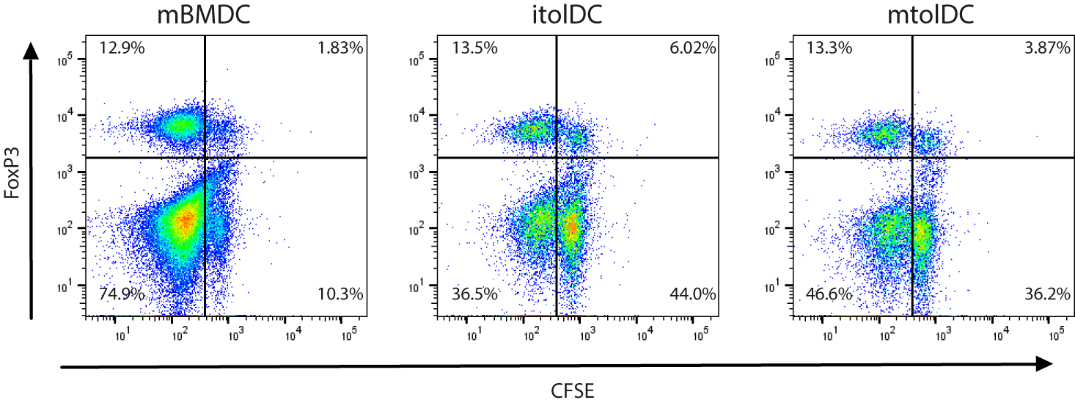
**

**Supplementary Figure S4.**

First, hPG TCR transgenic CD4^+^Thy1.1^+^ T cells were activated in vivo by injecting hPG peptide i.m. into the transgenic mice. After three days, CD4+ Thy1.1+ TCR transgenic T cells were isolated and transferred into a naïve Balb/c acceptor mouse. One day later, hPG pulsed (im)mature tolDCs or mature BMDCs were injected into the acceptor mice. After three days, phenotype and proliferation of the transferred CD4^+^Thy1.1^+^ T cells was measured by flow cytometry in spleen Representative plots of CSFE dilution and FoxP3 expression are shown.

**
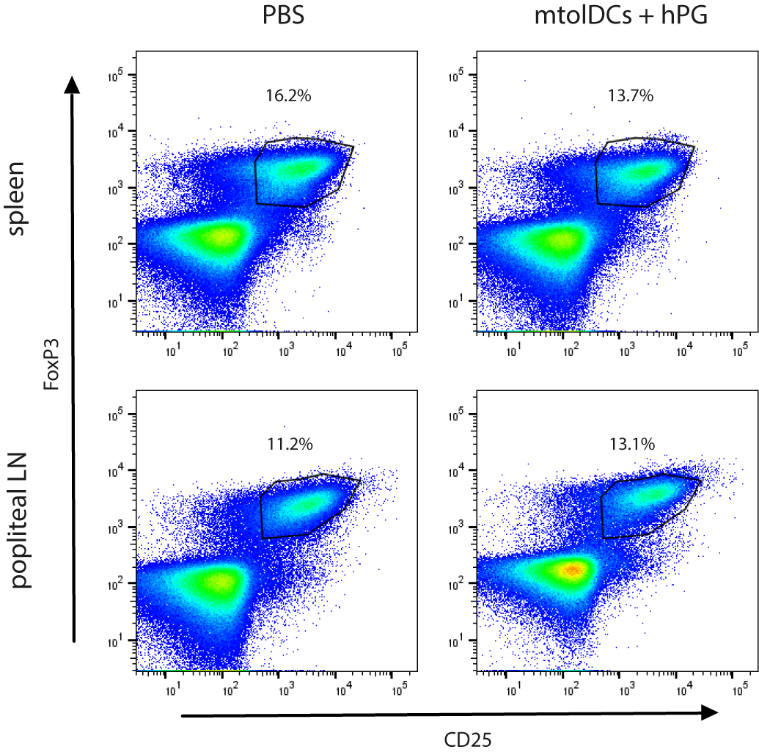
**

**Supplementary Figure S5.**

The percentage CD4^+^CD25^+^FoxP3^+^  was analyzed *ex vivo* in the spleen and popliteal lymph nodes by flow cytometry in arthritic mice treated with PBS or mtolDCs loaded with hPG as described in Materials and Methods. Arthritis was induced by injecting the mice two times (day 0 and day 21) with hPG protein and DDA. mTolDCs loaded with hPG (1x10^6^ cells in 200 µL PBS) were administered intravenously on day 17, before the second hPG/DDA injection. Spleens and lymphnodes were isolated at day 56 of the experiments and analyzed by flowcytometry. PBS: n=6, mtolDCs + hPG: n=7. Representative dotplots are shown. Doplots of mice receiving mtolDCs no ag ( n=5) were comparableto mtolDC + hPG (data not shown.

**
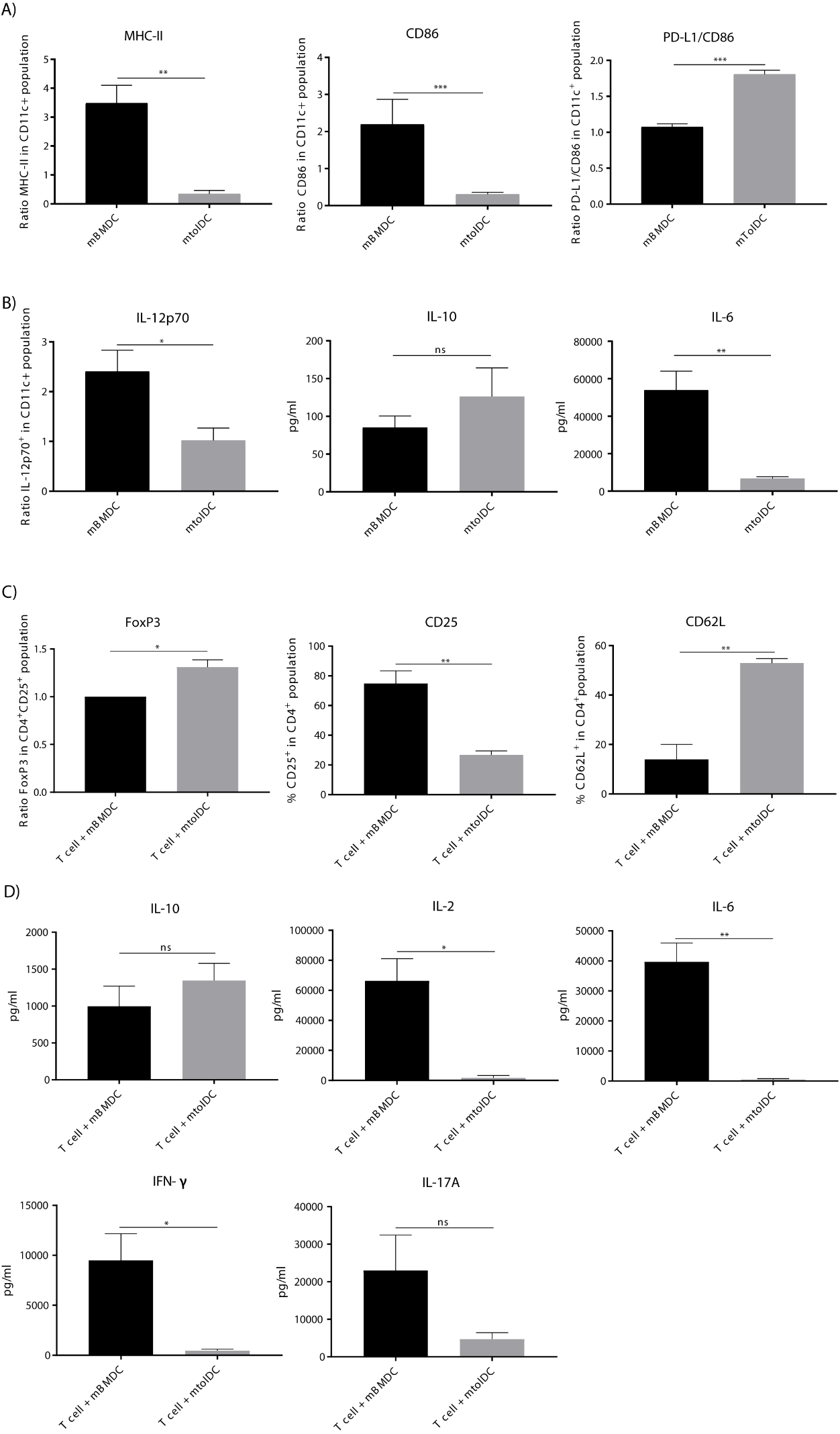
**

**Supplementary Figure S6.**

*LPS stimulated tolDCs show semi-mature phenotype.*

TolDCs were generated by adding dexamethasone and 1,25-dihydroxyvitamin D3 and stimulated with LPS. Phenotype was measured by flow cytometry **(A)** and the ratio to immature BMDC was used to determine the difference in expression of MHC-II, CD86 and PD-L1. Cytokine production **(B)** was measured in the supernatant by Magpix (IL-10, IL-6, GMCSF) or intracellular by flow cytometry (IL-12p70). MBMDCs or mtolDCs were pulsed with peptide and co-cultured for three days with naïve (CD25^+^ and CD44^+^ depleted) CD4^+^ T cells. On day 3, phenotype of the CD4^+^ T cells was determined by flow cytometry **(C)**. For FoxP3, the ratio to CD4^+^ T cells that were in co-culture with BMDCs was used to compare the difference when co-culturing with tolDCs. As markers for activation status of the CD4^+^ T cell, CD25 and CD62L were measured. Cytokine production was measured after co-culture in the supernatant by Magpix **(D)**. Two-tailed paired student T-test was used. *p≤0.05, **p≤0.01, ***p≤0.001. N = 4
